# A life LINE for large viruses

**DOI:** 10.7554/eLife.83488

**Published:** 2022-10-25

**Authors:** Eugene V Koonin, Mart Krupovic

**Affiliations:** 1 National Center for Biotechnology Information, National Institutes of Health Bethesda United States; 2 Institut Pasteur, Université Paris Cité, CNRS UMR6047, Archaeal Virology Unit Paris France

**Keywords:** vaccinia virus, horizontal gene transfer, evolution, poxvirus, LINE-1, retrotransposons, Human, Viruses

## Abstract

As long suspected, poxviruses capture host genes through a reverse-transcription process now shown to be mediated by retrotransposons.

**Related research article** Rahman MJ, Haller SL, Stoian AMM, Li J, Brennan G, Rothenburg S. 2022. LINE-1 retrotransposons facilitate horizontal gene transfer into poxviruses. *eLife* **11**:e63327. doi: 10.7554/eLife.63327.

**Related research article** Fixsen SM, Cone KR, Goldstein SA, Sasani TA, Quinlan AR, Rothenburg S, Elde NC. 2022. Poxviruses capture host genes by LINE-1 retrotransposition. *eLife* **11**:e63332. doi: 10.7554/eLife.63332.

Viruses with double-stranded DNA genomes have been stealing genes from their hosts for billions of years. In fact, these acquisitions represent the major force in viral evolution, with some ancient gene gains apparently underlying the emergence of the main groups of viruses we know today (Caprari et al., 2015; Koonin et al., 2022). Captured genes are often involved in the complex interactions between a virus and its specific host, leading to virus families having dramatically different repertoires of stolen genes (Elde and Malik, 2009; Koonin et al., 2022).

Herpesviruses and poxviruses are the two most common groups of double-stranded DNA viruses that infect animals. While herpesviruses reproduce in the nucleus and poxviruses in the cytoplasm, both have captured and then repurposed numerous host genes for counter-defense. In particular, the genome of orthopoxviruses – a group that includes the smallpox, monkeypox and cowpox viruses – contains about 100 captured genes that primarily help the viruses to evade the immune system (Moss and Smith, 2021). These sequences were acquired in batches over the 500 million years of evolution which separate fish poxviruses from the sub-family which includes orthopoxviruses (Senkevich et al., 2021).

Despite the importance of this phenomenon, exactly how double-stranded DNA viruses capture host genes remains unclear. In principle, two routes appear possible: virus and host could exchange segments of their genomes in the nucleus via a process known as recombination; or a messenger RNA (mRNA) from the host could be reverse-transcribed into DNA that is then integrated into the viral genome. This second mechanism, however, would require external assistance since neither poxviruses nor herpesviruses encode the enzymes responsible for reverse transcription.

This help could come from retrotransposons, the widespread mobile elements that can replicate and insert themselves into new locations in the genome of eukaryotes. In particular, retrotransposons such as LINE-1 (an element common in vertebrate genomes) are transcribed into an mRNA molecule which is then translated in the cytoplasm. The produced proteins import the LINE-1 RNA back into the nucleus and reverse transcribe it into DNA, concomitantly inserting it into new genomic locations (Figure 1A). DNA viruses could co-opt this process to turn host mRNAs into DNA that is incorporated into viral genomes.

**Figure 1.**
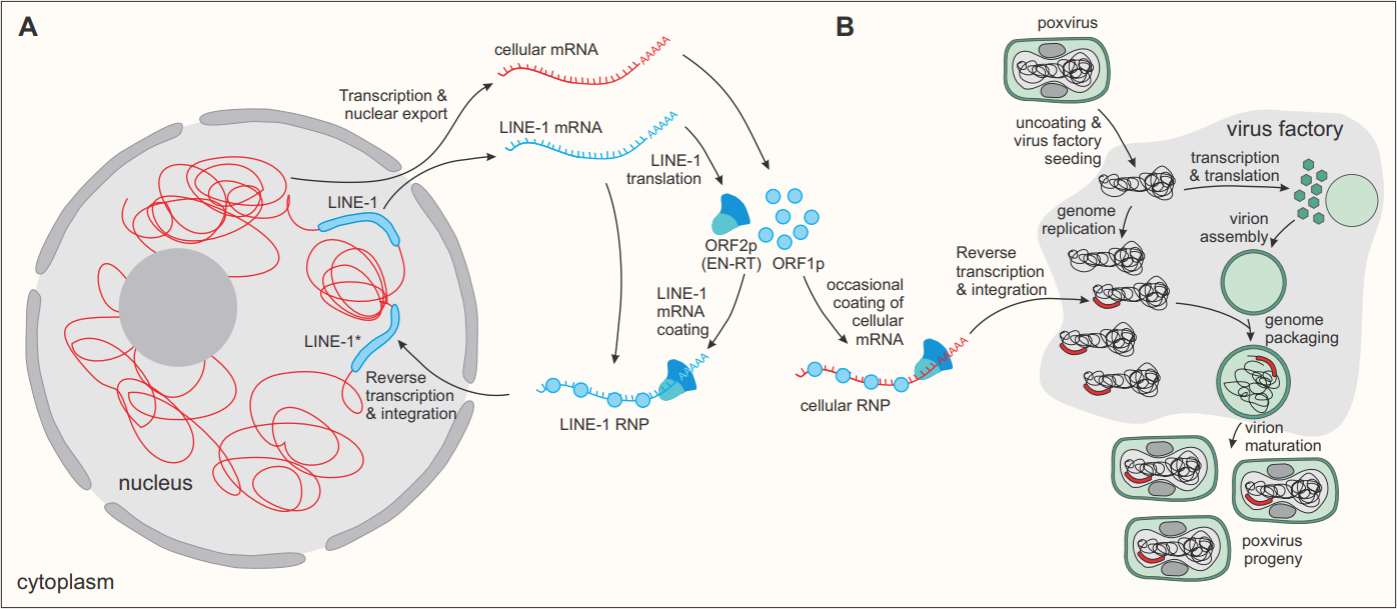
The reverse-transcription route of host gene capture by poxviruses. (**A**) LINE-1 (blue) is a retrotransposon commonly present in mammalian genomes (red). The LINE-1 DNA sequence already embedded in a chromosome is transcribed into a mature, polyadenylated LINE-1 mRNA (blue transcript), which is exported from the nucleus and translated to produce ORF1p and ORF2p (blue shapes). These two proteins bind to the LINE-1 mRNA (resulting in a LINE-1 ribonucleoprotein or RNP complex), import it back to the nucleus and then reverse-transcribe it into a DNA segment that is re-integrated into the genome. This results in a new copy of the retrotransposon (LINE-1*). (**B**) Poxviruses can use this reverse transcription mechanism to capture host genes. The ORF1p and ORF2p produced by LINE-1 occasionally form a complex with cellular mRNAs (red); the resulting cellular RNP complexes can undergo reverse-transcription and integration into target DNA. Upon infection, poxviruses establish cytoplasmic virus factories, where virus genome replication and virion assembly occur. Cellular mRNA RNPs apparently find their way into the poxvirus factories, where they get reverse-transcribed and inserted into the virus genome.

Two strong arguments support the reverse-transcription route. First, all viral genes lack introns (with a single exception in the herpesvirus family). These non-coding sequences are present within cellular genes but are removed from mature mRNA molecules – suggesting that host genes are acquired through an mRNA-mediated process rather than through DNA material being exchanged via recombination in the nucleus. Second, the cellular machinery that makes replication and recombination possible is only present in the nucleus; it is therefore inaccessible to poxviruses, which multiply in the cytoplasm. It has thus been hypothesized that poxviruses capture host genes via a reverse transcription-dependent mechanism (Lefkowitz et al., 2006). Yet virtually no specific evidence has been available to corroborate this conjecture so far. Now, in eLife, two teams based in the United States – one led by Nels Elde, with Sarah Fixsen as first author, and one led by Stefan Rothenburg with M Julhasur Rahman as first author – finally report such evidence (Fixsen et al., 2022; Rahman et al., 2022).

Dissecting how poxviruses capture host genes requires accelerating evolutionary processes that normally occur on time scales incommensurate with human lifetimes. Rahman et al. managed this feat by engineering specific, strong selective pressures. They focused on the vaccinia virus, an orthopoxvirus that carries two genes (E3L and K3L) which allow it to escape a host defence mechanism mediated by PKR, a DNA-dependent protein kinase. PKR-sensitive vaccinia virus mutants that lacked both E3L and K3L were created and introduced into human cells expressing a cloned copy of the viral E3L. After screening hundreds of millions of clones, 20 PKR-resistant variants which had acquired the E3L gene from their host were identified.

Crucially, the cells carried an E3L construct which included an engineered intron: this allowed the team to spot whether the viruses had acquired the gene through DNA-level recombination (which would preserve the intron), or through a reverse transcription mechanism (which would remove it). The intron was spliced out in all 20 PKR-resistant clones, pointing to the reverse transcription-dependent gene capture route. A closer look revealed that inserted E3L genes contained genetic hallmarks associated with LINE-1 activity, suggesting that the retrotransposon had catalyzed their integration into the viral genome (Figure 1B).

The evolutionary experiments by Fixsen et al., which focused on the K3L gene rather than E3L, yielded similar results. Remarkably, complementary evidence also exists from previous studies which had examined natural variants of other orthopoxviruses. Indeed, a recently captured gene retained the same tell-tale LINE-1 signatures in a strain of cowpox virus; and the genome of the taterapox virus carries signals from SINE retrotransposons, which depend on LINE-1 for their genomic insertion (Gubser et al., 2007; Piskurek and Okada, 2007).

Large cytoplasmic DNA viruses have long been suspected to capture host genes through a reverse-transcription route, yet comparative genomics approaches had been unable to convincingly identifying the mechanism at play. And indeed, the signatures of LINE-1-mediated insertion quickly decay during evolution, making them difficult to identify by genome comparison. Instead, ingeniously designed evolutionary experiments came to the rescue. As these methods get refined, we can expect to uncover similar evolutionary mechanisms in other viruses, and even perhaps alternative strategies for capturing host genes. Beyond the implications for poxvirus evolution, the experiments of Rahman et al. and Fixsen et al. demonstrate that LINE-1 elements can be active not only in the nucleus, but also in the cytoplasm of mammalian cells. The consequences of such activity remain to be investigated.
